# Cardiac Myxoma Presenting With Multiple Ischemic Strokes and Critical Limb Ischemia: A Case Report

**DOI:** 10.7759/cureus.105952

**Published:** 2026-03-27

**Authors:** Manal Touilite, Soumia Ait Ami, Mohamed Chraa, Nissrine Louhab

**Affiliations:** 1 Department of Neurology, Mohamed VI Hospital University of Marrakesh, Cadi Ayyad University, Marrakesh, MAR

**Keywords:** cardiac myxoma, case report, critical limb ischemia, infective endocarditis, ischemic stroke, peripheral artery disease, systemic embolism

## Abstract

Left atrial myxomas are rare, benign cardiac tumors that are often asymptomatic but may present with systemic embolic events. We report the case of a middle-aged man who presented with acute ischemic stroke, which led to the diagnosis of a left atrial myxoma. Further evaluation revealed associated infective endocarditis and critical limb ischemia, secondary to systemic embolization. The patient underwent successful surgical excision of the tumor, with multidisciplinary management.

This case highlights the importance of considering cardiac sources in embolic stroke of unclear origin and underscores the need for prompt diagnosis and coordinated care to prevent severe complications.

## Introduction

Cardioembolic strokes account for approximately 20% of acute ischemic strokes and are associated with a high risk of early recurrence and mortality [1]. When the clinical presentation suggests an embolic mechanism, a thorough evaluation to identify a potential cardiac source is essential for appropriate management [1]. Among the various etiologies, cardiac myxoma represents a rare but potentially curable cause of cardioembolic stroke.

Cardiac myxoma is the most common primary cardiac tumor in adults, accounting for 50%-85% of benign cardiac neoplasms [2]. It most frequently arises in the left atrium (60%-80% of cases), typically originating from the region of the fossa ovalis [2]. While small tumors may remain asymptomatic, larger lesions can lead to a wide spectrum of clinical manifestations, including obstructive cardiac symptoms, systemic embolization, and constitutional inflammatory features [2]. Peripheral embolism represents the initial manifestation in approximately 16% of cases, and may occur during the course of the disease in up to 33% of patients [3].

Although cardiac myxoma is a recognized cause of cardioembolic stroke, its presentation with the simultaneous occurrence of recurrent ischemic stroke, peripheral arterial embolization, and superimposed infective endocarditis remains exceptionally rare and represents a particularly severe clinical scenario. Such an association poses significant diagnostic and therapeutic challenges, often requiring urgent and coordinated, multidisciplinary management. This case is reported to expand the clinical spectrum of cardiac myxoma and to emphasize the importance of early recognition of atypical, multi-system presentations in order to optimize patient outcomes.

## Case presentation

A 50-year-old male, with no prior history of hypertension, diabetes, or tobacco use, presented in April 2024 with sudden weakness of the left side of the body, difficulty speaking, and facial asymmetry. He sought medical attention approximately 30 hours after symptom onset, following a generalized tonic-clonic seizure and a decreased level of consciousness. The patient was initially admitted to the intensive care unit and later transferred to our department for further evaluation and management.

**Figure 1:**
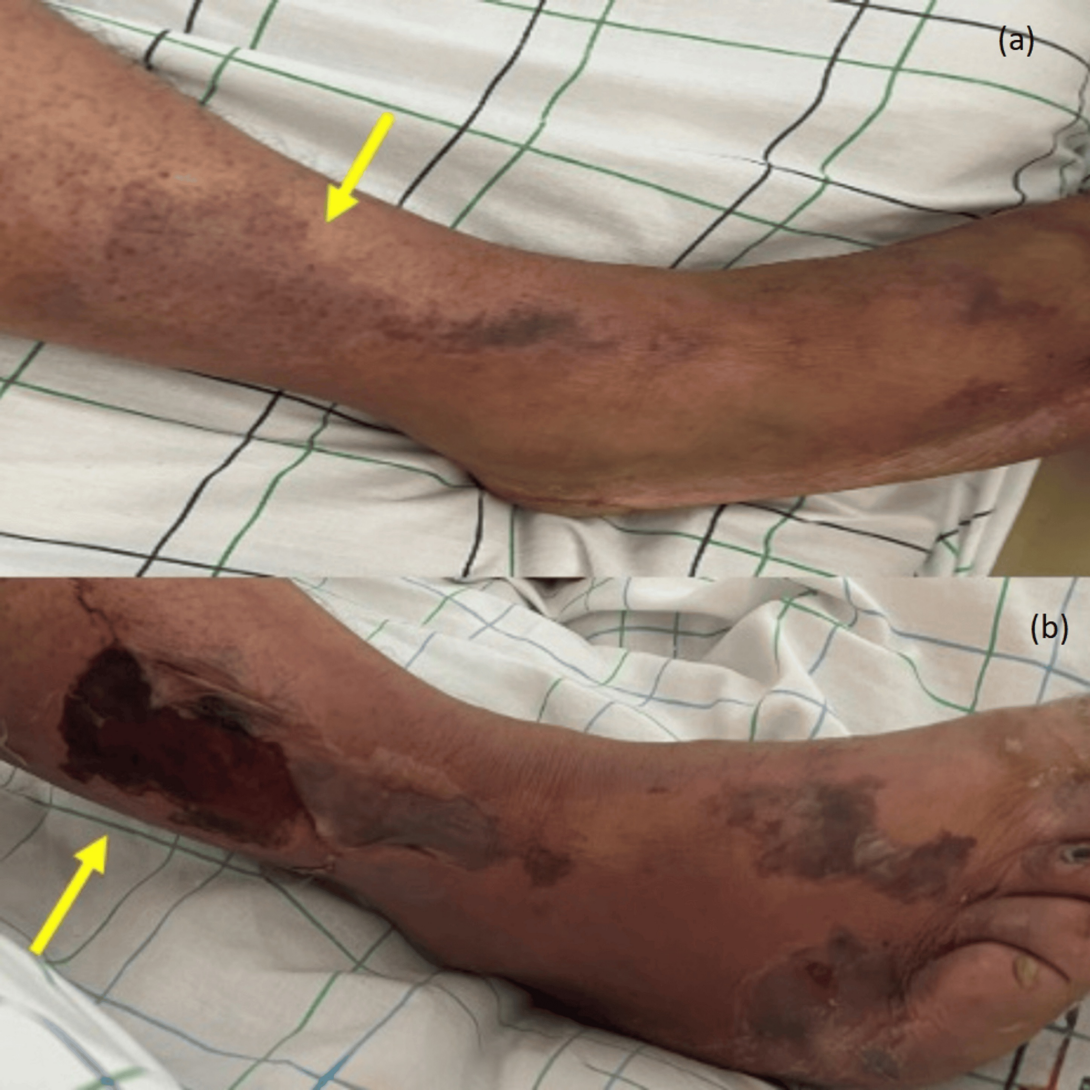
Clinical photograph of the right leg (a) Clinical photograph of the right leg, showing an erythematous, violaceous, cold, and tender patch on the anterolateral aspect, consistent with peripheral embolism (yellow arrow). (b) Progression of the cutaneous findings after 72 hours (yellow arrow).

On examination, he was in good general condition, with a normal body mass index. Neurological assessment revealed a left pyramidal syndrome, with mild lower limb weakness, increased muscle tone, exaggerated reflexes, and left plantar extension. The patient also reported pain in the anterolateral aspect of the right leg, which was erythematous, violaceous, cold, and tender, suggestive of peripheral embolism (Figure 1). Vital signs included a heart rate of 87 bpm and a blood pressure of 135/80 mmHg.

**Figure 2:**
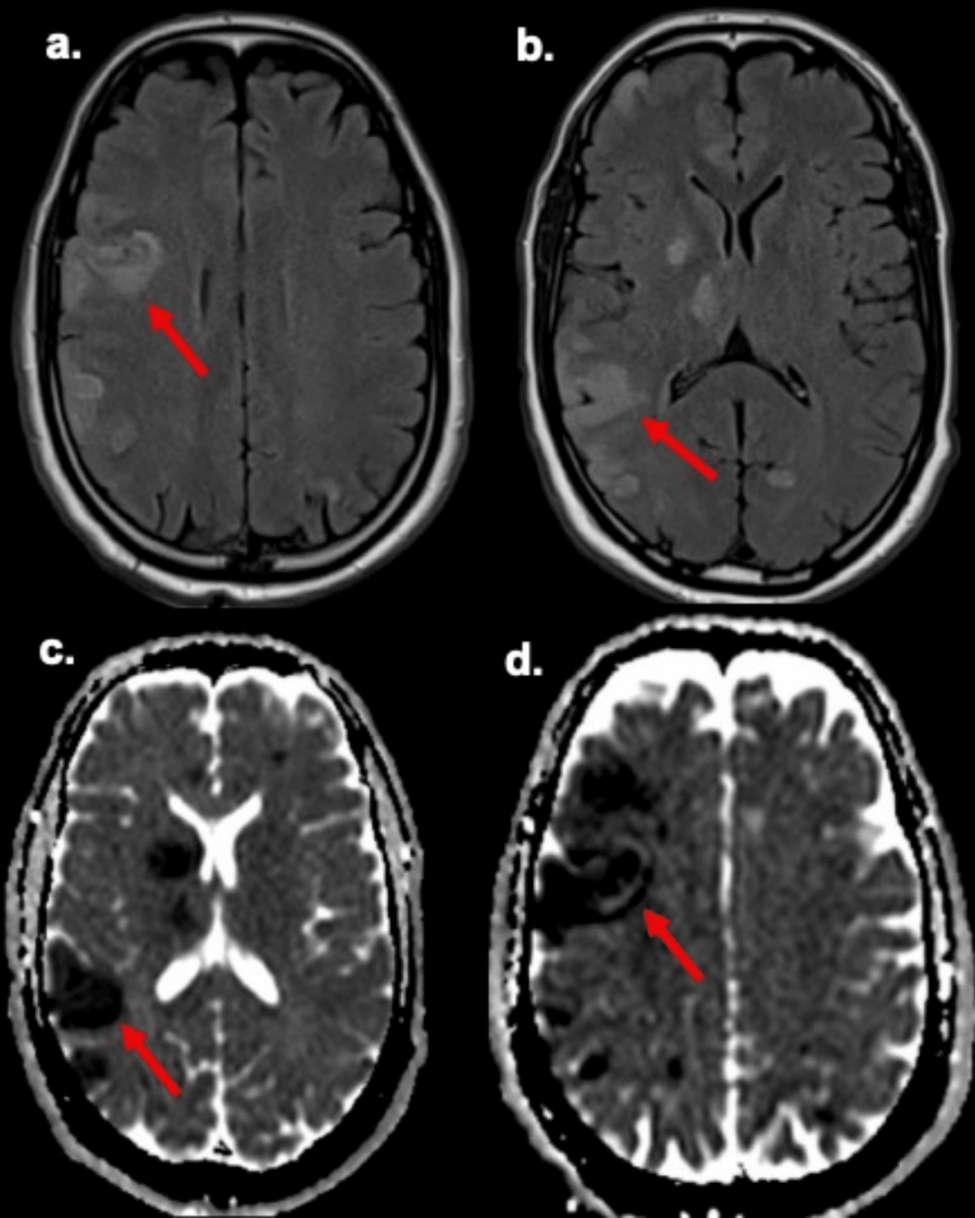
MRI of the brain (a-b) Brain MRI showed multiple hyperintense lesions on FLAIR sequences, involving the right cerebral hemisphere and the thalamolenticular region, consistent with multiple ischemic infarcts (red arrow). (c-d) Corresponding ADC maps show restricted diffusion, with low signal on ADC maps (red arrow). MRI: magnetic resonance imaging; FLAIR: fluid-attenuated inversion recovery; ADC: apparent diffusion coefficient

Brain magnetic resonance imaging (MRI) demonstrated multiple bilateral ischemic strokes, some with hemorrhagic transformation, consistent with a cardioembolic pattern (Figure 2). Antiplatelet therapy with aspirin and high-intensity statin therapy with atorvastatin were initiated as part of secondary stroke prevention. The electrocardiogram showed sinus rhythm without chamber enlargement or significant conduction abnormalities. During hospitalization, the patient developed a fever up to 40 °C. Laboratory tests revealed leukocytosis and elevated inflammatory markers (erythrocyte sedimentation rate (ESR) 33 mm/h and C-reactive protein (CRP) 377 mg/L). Blood cultures grew multidrug-resistant *Enterococcus faecium*, sensitive only to teicoplanin, prompting initiation of intravenous antibiotic therapy. Teicoplanin was administered at a loading dose of 6 mg/kg every 12 hours for three doses, followed by 8 mg/kg once daily. The total duration of therapy was 42 days, with therapeutic drug monitoring and close clinical and microbiological follow-up, resulting in rapid clinical improvement.

**Figure 3:**
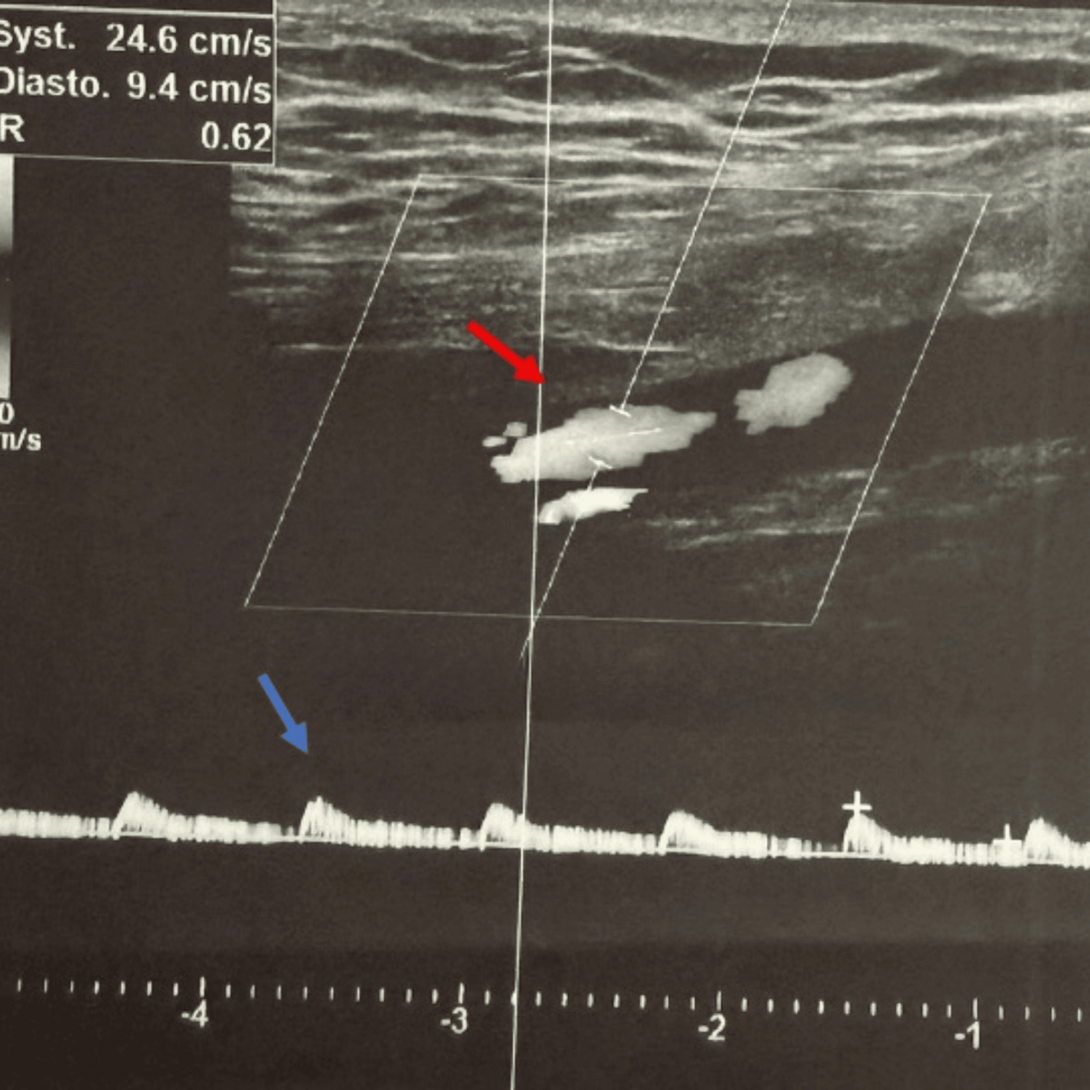
Doppler ultrasound of the right lower limb Doppler ultrasound of the right lower limb showed total occlusion of the right popliteal artery (red arrow), and dampened flow in distal arteries (blue arrow).

**Figure 4:**
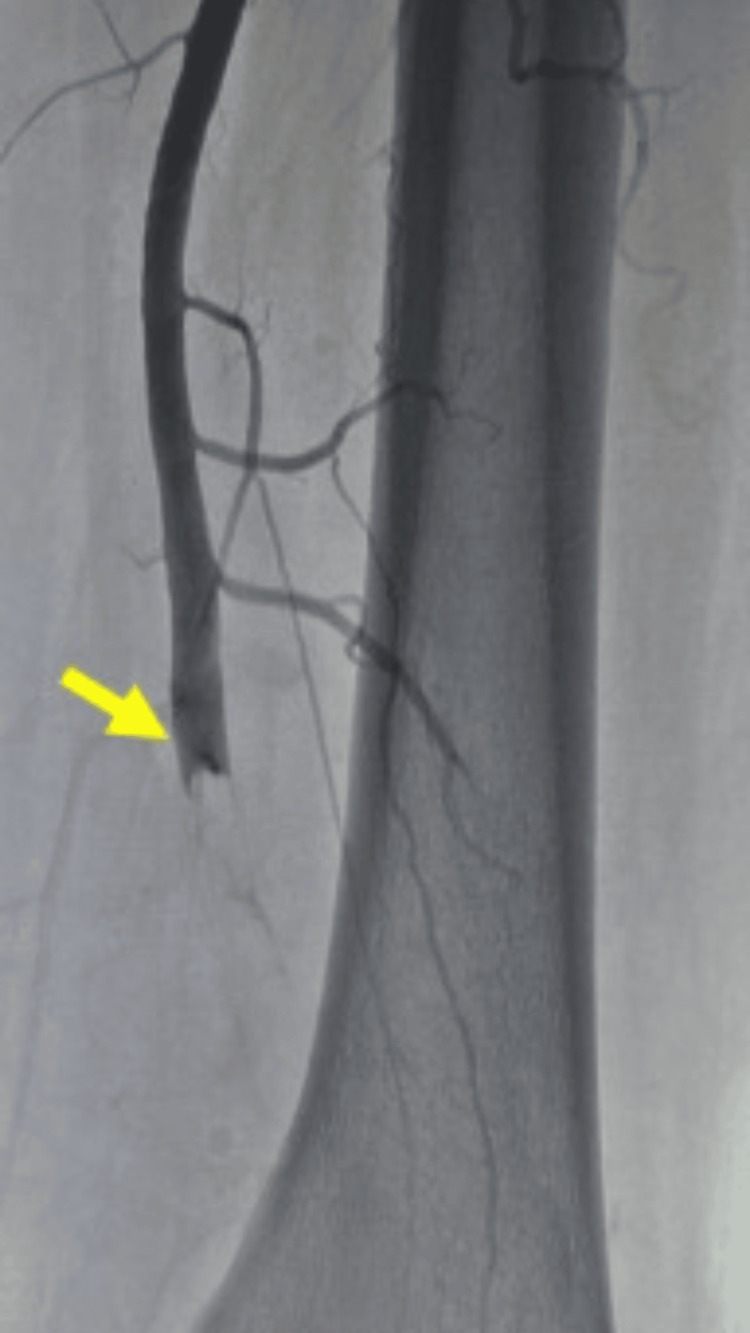
Arteriography of the right lower limb Arteriography of the right lower limb demonstrated thrombosis of the popliteal artery at the articular segment, with absence of distal recanalization (yellow arrow).

Transthoracic echocardiography revealed a large, mobile, heterogeneous mass in the left atrium (61 × 28 mm), attached to the interatrial septum and extending toward the anterior mitral leaflet, consistent with an atrial myxoma. Left ventricular systolic function was preserved (ejection fraction 60%), with mild left atrial enlargement. Meanwhile, the patient developed signs of acute limb ischemia, characterized by persistent pain and features suggestive of threatened limb viability. Doppler ultrasound and arteriography of the right lower limb confirmed arterial occlusion (Figures 3-4). Intravenous unfractionated heparin was promptly initiated, with a bolus dose of 70 IU/kg, followed by a continuous infusion of 12 IU/kg/hour. The infusion rate was adjusted to maintain an activated partial thromboplastin time (aPTT) at 1.5-2.5 times the control value, and treatment was continued for four days to prevent thrombus propagation and further embolic events.

Given the severity of limb ischemia and the presence of an intracardiac embolic source, urgent surgical management was undertaken. Under general anesthesia, the patient first underwent right lower limb embolectomy using a Fogarty catheter, allowing restoration of arterial flow. This was followed by median sternotomy and complete surgical excision of the left atrial mass under cardiopulmonary bypass. The tumor was successfully removed, along with its attachment to the interatrial septum. Histopathological analysis revealed stellate and polygonal cells embedded in a myxoid stroma, consistent with cardiac myxoma, confirming the diagnosis.

Postoperatively, the patient demonstrated significant neurological recovery and complete resolution of limb ischemia. On postoperative day 5, following confirmation of adequate hemostasis, anticoagulation was transitioned to oral therapy with rivaroxaban at 15 mg twice daily for 21 days, followed by 20 mg once daily for three months, given the embolic risk in the postoperative context. After completing the three-month course of rivaroxaban, anticoagulation was discontinued and replaced with aspirin for long-term secondary prevention, alongside atorvastatin. Follow-up evaluations at one, three, and six months showed that the patient remained clinically stable, with no evidence of recurrent embolic events or tumor recurrence, and continued to demonstrate good functional recovery.

## Discussion

Primary cardiac tumors are rare, with an estimated incidence of approximately 1,380 cases per 100 million individuals [4]. Atrial myxoma is the most common primary cardiac tumor and accounts for nearly half of benign cardiac neoplasms [4]. Although histologically benign, its marked mobility and friability confer a substantial embolic risk, as tumor fragments or superimposed thrombi may enter the systemic circulation and cause severe ischemic complications [5]. This embolic potential explains why atrial myxoma is often regarded as a “great imitator,” with clinical manifestations typically classified as constitutional, obstructive, or embolic [6]. In our patient, multiple bilateral ischemic infarcts on brain imaging strongly suggested a cardioembolic source [1]. The associated peripheral arterial embolism, manifested by right leg pain, coldness, and Doppler-confirmed occlusion, further supported the diagnosis of systemic embolization. Extracranial embolic events have been reported in approximately 10%-30% of patients with cardiac myxoma [3]. The coexistence of cerebral and peripheral ischemia in the same patient, although uncommon, has been described in case reports and underscores the heterogeneity of embolic manifestations associated with mobile left atrial masses [7].

Transthoracic echocardiography remains the first-line diagnostic tool and usually demonstrates a mobile, heterogeneous intracardiac mass, attached to the interatrial septum [6]. Transesophageal echocardiography may further define tumor morphology and attachment, and is particularly useful when transthoracic views are suboptimal [7]. In the present case, transthoracic echocardiography identified a 61 × 28 mm lesion consistent with atrial myxoma, in keeping with the typical size range described in larger series, often 4-6 cm in maximal diameter [7].

Once diagnosed, prompt surgical resection remains the treatment of choice to prevent recurrent embolic events, which may occur in about 10% of patients while awaiting surgery [8]. However, the timing of surgery after acute ischemic stroke remains controversial. Some authors recommend delaying surgery for one to two weeks after a major stroke to reduce the risk of hemorrhagic transformation, particularly in severe neurological deficits, whereas others advocate earlier intervention in the presence of recurrent or life-threatening embolization [9]. In our case, urgent surgery was undertaken after stabilization of the acute neurological status and control of infective features, with successful resection and good neurological recovery. This approach is consistent with recent multidisciplinary strategies that support individualized timing, based on clinical severity rather than a fixed delay [9].

Comparison with previously reported cases reveals both similarities and distinctive features. As in many published reports, our patient was relatively young and lacked traditional cardiovascular risk factors, consistent with the observation that cardioembolic events in myxoma often occur in patients without common stroke risk profiles [7]. However, the concurrent triple manifestation - multiple cerebral infarctions, acute limb ischemia, and superimposed infection - has rarely been reported. When infection is present, it is more commonly associated with typical microorganisms, such as *Staphylococcus aureus*, rather than multidrug-resistant enterococci [10]. The identification of multidrug-resistant *E. faecium* as the causative organism in our patient further distinguishes this case from more typical myxoma-related presentations and suggests either bacteremic seeding or an unusually aggressive infective process. This observation raises the possibility that, although embolic complications in myxoma are well recognized, the coexistence of embolic and infective manifestations may represent a distinct clinical phenotype, requiring heightened suspicion and prompt multidisciplinary management.

The pathophysiological basis for these manifestations is likely multifactorial. The embolic potential of cardiac myxomas is thought to result from tumor friability and mobility, allowing fragmentation and systemic embolization [5]. A superimposed infection may further increase embolic risk by inducing local inflammation, endothelial injury, and a procoagulant state. Multidrug-resistant organisms may complicate this interplay by prolonging bacteremia and promoting thrombogenesis. These mechanistic considerations highlight the need for early diagnosis, aggressive antimicrobial therapy when indicated, and timely surgical management to mitigate both embolic and infective sequelae [10].

Cardiac myxoma should be considered in young patients presenting with embolic stroke, particularly when multiple vascular territories are involved, or when unexplained systemic inflammatory features are present [11]. The rare combination of recurrent cerebral infarction, acute peripheral embolism, and infective features represents both a diagnostic challenge and a therapeutic priority. This case underscores the importance of multidisciplinary care and heightened vigilance for atypical presentations of cardiac tumors.

## Conclusions

In conclusion, atrial myxoma is a rare but important cause of embolic stroke, and may present with nonspecific or misleading clinical manifestations, making diagnosis challenging. This case highlights the value of a thorough clinical evaluation and early transthoracic echocardiography in patients presenting with multi-territory or systemic embolic events. Given the high risk of recurrent embolization and sudden cardiac complications while awaiting treatment, urgent surgical resection remains the definitive management to achieve optimal outcomes.
